# Draft Genome Sequence of Acid-Tolerant *Clostridium drakei* SL1^T^, a Potential Chemical Producer through Syngas Fermentation

**DOI:** 10.1128/genomeA.00387-14

**Published:** 2014-05-15

**Authors:** Yujin Jeong, Yoseb Song, Hyeon Seok Shin, Byung-Kwan Cho

**Affiliations:** a Department of Biological Sciences and KAIST Institute for the BioCentury, Korea Advanced Institute of Science and Technology, Daejeon, South Korea; b Intelligent Synthetic Biology Center, Daejeon, South Korea

## Abstract

*Clostridium drakei* SL1^T^ is a strictly anaerobic, H_2_-utilizing, and acid-tolerant acetogen isolated from an acidic sediment that is a potential platform for commodity chemical production from syngas fermentation. The draft genome sequence of this strain will enable determination of the acid resistance and autotrophic pathway of the acetogen.

## GENOME ANNOUNCEMENT

Acetogenic bacteria produce over 10^12^ kg of acetic acid annually, which comprises 10% of the global acetate production (1). In this process, acetogens autotrophically reduce CO_2_ to acetate with H_2_ as the sole electron donor through the reductive acetyl coenzyme A (acetyl-CoA) pathway, also known as the Wood–Ljungdahl pathway (2). In addition, CO, one of the most abundant syngases, can be utilized for autotrophic growth as the sole electron donor. Because of these metabolic capabilities, acetogens are an attractive platform for the production of useful multi-carbon compounds by syngas fermentation. Among acetogenic clostridial species, *Clostridium drakei* SL1^T^ is a unique acetogen isolated from the sediment of an acidic coal mine pond (3). Moreover, to date, most of the acetogens have been isolated from a habitat with neutral pH, which suggests that an acidic environment is unfavorable for acetogens (4). Further, *C. drakei* utilizes a wide range of substrates such as glucose, fructose, arabinose, and xylose to produce acetate as the main reduced end product (3). To understand the unique physiological properties, including acid resistance, and metabolic capabilities of syngas utilization of *C. drakei*, we obtained its genome sequence information.

The *C. drakei* genome was sequenced using Illumina MiSeq with a 2×150-cycle paired-end platform (Illumina, San Diego, CA). The sequencing library was constructed using a TruSeq DNA sample prep kit (Illumina). Before sequence assembly, phiX sequences were removed and the reads were trimmed by their qualities (low-quality sequence, 0.05 limit, and ambiguous nucleotides, maximum of 2 nucleotides allowed) using CLC Genomics Workbench (CLC Bio, Aarhus, Denmark). We obtained a total of 4,441,384,356 bases in 30,418,087 reads, which were then assembled using CLC Genomics Workbench (minimum contig length, 302; automatic bubble size, yes; word size, 61; perform scaffolding, yes). The assembly resulted in 140 contigs, the largest of which is 1,132,561 bases. The draft genome sequence was annotated using the RAST server (5).

The resulting draft genome sequence of *C. drakei* comprises 5,635,531 bases, with a 35% G+C content and 5,763 predicted protein-coding sequences (CDSs). Eleven rRNAs and 100 tRNAs were predicted using the RNAmmer 1.2 server (6) and tRNAscan-SE 1.23 (7), respectively. The genome of *C. drakei* is larger than those of other acetogenic clostridial species such as *C. ljungdahlii* (4.6 Mbp) (8) and *Moorella thermoacetica* (2.6 Mbp) (2) but smaller than those of *C. ultunense* Esp (6.2 Mbp) (9) and *C. methoxybenzovorans* (7.0 Mbp) (accession number ATXD00000000). *C. drakei* possesses the metabolic pathway required to produce butanol from acetyl-CoA, which is mediated by the NADH-dependent butanol dehydrogenase. *C. acetobutylicum* and *C. carboxidivorans* (5.6 Mbp), which is closely related to *C. drakei*, also possess this enzyme (10). An in-depth study of the metabolic pathway would enable application of *C. drakei* as a potential industrial platform for utilization of cellulose-derived sugars such as xylose, arabinose, and rhamnose (11).

### Nucleotide sequence accession numbers.

The draft genome sequence of *C. drakei* has been deposited at the DDBJ/EMBL/GenBank database under the accession no. JIBU00000000. The version described in this paper is the first version, JIBU01000000.
