# Correction for Jeong et al., Draft Genome Sequence of Acid-Tolerant *Clostridium drakei* SL1^T^, a Potential Chemical Producer through Syngas Fermentation

**DOI:** 10.1128/genomeA.01465-14

**Published:** 2015-02-12

**Authors:** Yujin Jeong, Yoseb Song, Hyeon Seok Shin, Byung-Kwan Cho

**Affiliations:** a Department of Biological Sciences and KAIST institute for the BioCentury, Korea Advanced Institute of Science and Technology, Daejeon, South Korea; b Intelligent Synthetic Biology Center, Daejeon, South Korea

## AUTHOR CORRECTION

Volume 2, no. 3, e00387-14, 2014. Page 1, column 1, lines 26 and 27: “and ambiguous nucleotides, maximum of 2 nucleotides allowed” should read “and no ambiguous nucleotide allowed.”

 Page 1, column 1, line 29: “4,441,384,356 bases in 30,418,087 reads” should read “2,282,553,711 bases in 15,225,954 reads.”

 Page 1, column 1, lines 32 and 33: “resulted in 140 contigs, the largest of which is 1,132,561 bases” should read “resulted in 122 contigs, the largest of which is 470,943 bases.”

 Page 1, column 1, lines 36 and 37: “5,635,531 bases, with a 35% G+C content and 5,763 predicted protein-coding sequences (CDSs)” should read “5,578,774 bases, with a 35% G+C content and 5,094 predicted protein-coding sequences (CDSs).”

 Page 1, column 1, line 37: “Eleven rRNAs and 100 tRNAs” should read “six rRNAs and 74 tRNAs.”

 Page 1, column 2, line 18: “is the first version, JIBU01000000” should read “is the second version, JIBU02000000.”

